# Cardiac MRI segmentation of the atria based on UU-NET

**DOI:** 10.3389/fcvm.2022.1011916

**Published:** 2022-11-24

**Authors:** Yi Wang, Shu-Ting Li, Jing Huang, Qing-Quan Lai, Yi-Fan Guo, Yin-Hui Huang, Yuan-Zhe Li

**Affiliations:** ^1^Department of CT/MRI, The Second Affiliated Hospital of Fujian Medical University, Quanzhou, China; ^2^Department of Radiology, The First Affiliated Hospital of Zhejiang Chinese Medical University (Zhejiang Provincial Hospital of Traditional Chinese Medicine), Hangzhou, China; ^3^Department of Neurology, Jinjiang Municipal Hospital, Quanzhou, China

**Keywords:** cardiac MRI, deep deconvolution neural network, UU-NET, image segmentation, edge detection segmentation, threshold segmentation

## Abstract

**Background and objective:**

In today's society, people's work pressure, coupled with irregular diet, lack of exercise and other bad lifestyle, resulting in frequent cardiovascular diseases. Medical imaging has made great progress in modern society, among which the role of MRI in cardiovascular field is self-evident. Based on this research background, how to process cardiac MRI quickly and accurately by computer has been extensively discussed. By comparing and analyzing several traditional image segmentation and deep learning image segmentation, this paper proposes the left and right atria segmentation algorithm of cardiac MRI based on UU-NET network.

**Methods:**

In this paper, an atrial segmentation algorithm for cardiac MRI images in UU-NET network is proposed. Firstly, U-shaped upper and lower sampling modules are constructed by using residual theory, which are used as encoders and decoders of the model. Then, the modules are interconnected to form multiple paths from input to output to increase the information transmission capacity of the model.

**Results:**

The segmentation method based on UU-NET network has achieved good results proposed in this paper, compared with the current mainstream image segmentation algorithm results have been improved to a certain extent. Through the analysis of the experimental results, the image segmentation algorithm based on UU-NET network on the data set, its performance in the verification set and online set is higher than other grid models. The DSC in the verification set is 96.7%, and the DSC in the online set is 96.7%, which is nearly one percentage point higher than the deconvolution neural network model. The hausdorff distance (HD) is 1.2 mm. Compared with other deep learning models, it is significantly improved (about 3 mm error is reduced), and the time is 0.4 min.

**Conclusion:**

The segmentation algorithm based on UU-NET improves the segmentation accuracy obviously compared with other segmentation models. Our technique will be able to help diagnose and treat cardiac complications.

## Introduction

Recently, the worldwide prevalence of cardiovascular disease also gradually rise, according to world health organization in 2019 chart “cardiovascular disease risk, according to the world heart vascular disease prevalence in the rising trend, number of cases to exceed 500 million, 17.9 million people die each year from cardiovascular disease, first in all kinds of diseases (1). The public should pay attention to the prevention and treatment of cardiovascular diseases (2). Generally applied in clinical practice, for the diagnosis of cardiovascular disease, doctors usually only by observing the patients with cardiac image two-dimensional image of the fault, and according to the experience of the clinical cases of his past give treatment, the diagnosis for the dependence of subjective experience is bigger, and has led to bigger misjudgment. In addition, in some cases, to save resources and time, methods such as improving mri speed, imaging time, or spatial resolution are often adopted, but in such cases, a large amount of additional data is generated, which greatly reduces the efficiency of doctors to read images (3).

Medical image segmentation is to mark or extract a specific part of a medical image and apply it to other medical tasks. Therefore, the segmentation performance of medical images directly affects the development of other related technologies, such as three-dimensional reconstruction of human organs, registration of human parts, and localization of diseased or necrotic tissues. However, the development of medical image segmentation technology is also limited by many aspects. First, there are many kinds of imaging principles of medical image, and the quality of imaging is greatly affected by acquisition equipment and environment, that is, it is easy to be interfered by various noises. Therefore, it is difficult to generate a universal segmentation technique for all medical images. Secondly, according to the biological characteristics of human beings, the anatomical structure of each organ in human body is extremely complex, and there are significant differences between different ages of the same individual and individuals, which further increases the difficulty of segmentation (4). However, if medical image segmentation can be completed quickly and accurately, it will effectively improve the application of relevant imaging technology in clinical medicine.

The current development of computer field has added a possibility for the development of medical image segmentation. In 2012, artificial intelligence (AI), which focuses on computing power and deep learning, became the core technology of the next era (5). As an important branch of artificial intelligence technology, computer vision is developing very fast. In the 2015 ImageNet image recognition contest (6), computer recognition accuracy surpassed that of humans for the first time. Therefore, if the computer can be used to pre-process medical images to increase the efficiency of doctors or directly diagnose related diseases, it will be beneficial to improve the level of medical treatment.

Magnetic resonance (MRI) is a common heart imaging technique. In the early stage of the development of imaging technology, MRI has a slow imaging speed and is not suitable for the acquisition of cardiac images that require high imaging speed and resolution. However, with the development of medical image imaging technology, cardiac MRI has been able to obtain cardiac motion images by means of time-sharing acquisition in multiple cardiac systolic and diastolic cycles. MRI is the use of hydrogen atoms in the magnetic field in the human body to release energy information sampling technology, unlike conventional CT, MRI without ionizing radiation damage, imaging at the same time to be able to reflect the high contrast between different blood groups, is helpful to obtain clear heart anatomy structure (7). Therefore, MRI is recognized as the gold standard for evaluating cardiac function. Therefore, the image segmentation technology in this paper will be based on MRI imaging.

The left atrium is directly connected with the left ventricle and pulmonary veins. Its basic functions include channel function, contraction function and reserve function. Channel function refers to the early and middle stage of left ventricular diastole, when the left atrium sends blood from the pulmonary vein to the left ventricle. Systolic function refers to the active contraction of left atrial myocardium to provide blood for the left ventricle. Reserve function means that the systole of the left ventricle acts as a vessel for storing blood. In addition, the atrial structure directly maintains human atrial fibrillation (8), and the main reason for the poor clinical performance of atrial fibrillation is the lack of a deeper understanding of the underlying atrial anatomical structure (9). Therefore, understanding the atrial morphology and changes of patients with atrial fibrillation has certain reference value for understanding the clinical treatment of atrial fibrillation.

Manual segmentation of the left atrium by a medical expert does guarantee high accuracy, but it can take a lot of time for the expert to annotate an individual's data. The boring and long-term nature of labeling images will often lead to fluctuations in the state of labeling images and affect the accuracy of labeling, that is, the accuracy level is subjective. The scarcity of medical experts also leads to the lack of widespread application of left atrial segmentation. Therefore, it is necessary to adopt the means of computer field to solve this problem.

However, there are many schemes for ventricular image segmentation based on machine learning, such as deep belief network (DBN) (10), stack autocoding (SAE) (11), deep autoencoder (DAE) (12) and other technologies. However, these researches mainly focus on neuroimage analysis and have little effect on ventricular image segmentation. Using random forest method (13) and constrained Boltzmann machine (14) will lead to high computational complexity and unstable results. Therefore, the above methods have great limitations in the segmentation of ventricular images.

To solve this problem, we design a local image segmentation method based on U-NET. Based on the full convolutional neural network and the combination of deconvolution technology and deconvolution technology, a dual cavity scan image segmentation based on U-NET network is realized. At the same time, in order to avoid excessive model parameters, the number of convolutional filters is appropriately set according to the characteristics of MRI and the needs of the segmentation task. Experimental results show that the segmentation method of U-NET atrial image has better effect compared with several existing classification methods.

## Methodology

### Region growing

The basic idea of regional growth is that regions to be segmented have the same or similar properties within the region and different properties outside the region (15). The specific implementation steps are as follows:

1. Select a seed pixel for each region to be segmented.
2. All pixels in the field of the region where the seed pixel is located are judged according to certain growth criteria. If there are pixels that meet the growth criteria, all the pixels will be absorbed into the region where the seed pixels are, and the second step will be performed again.
3. When there is no pixel satisfying the growth criterion in the field of a region, the region no longer expands, that is to say, the region has grown.

**Figure 1:**
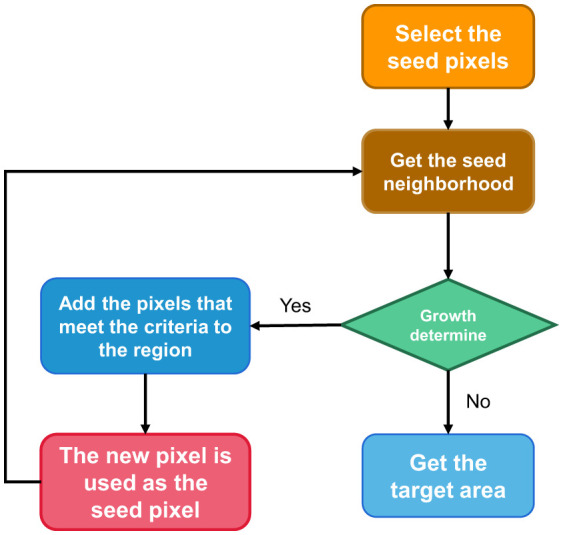
Region growth for image segmentation steps.

For the specific segmentation process, please refer to Figure 1, that is, select the growth cycle until the end of all growth.

It should be pointed out that the growth or similarity standard usually set is related to the characteristics of the image itself, usually, the difference between gray scale and gray scale is within a critical value. This paper uses a threshold of 25.

### Threshold segmentation method

#### Threshold segmentation

**Figure 2:**
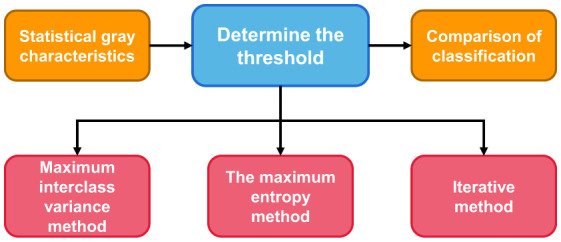
Steps of threshold segmentation method.

Threshold method were the first to be applied to achieve a segmentation method of image segmentation (16), the key technique of the threshold segmentation method is: according to the statistics of the whole image grayscale characteristics, according to the gray level range and the number of the gray value for that one or more of the image threshold value, then one by one traverse each gray value of the image, and each gray level values and threshold values After comparison, classify according to the final comparison results or preset rules, and divide each pixel within different threshold ranges into different categories in order to achieve segmentation. Figure 2 shows the general steps of the threshold segmentation method for image segmentation, among which there are many methods to determine the threshold.

#### The limitations method of threshold

##### Maximum interclass variance method

The maximum inter-class variance, also known as the algorithm, was proposed by The Japanese scholar Otsu. It is an algorithm for image binary segmentation threshold. The basic idea of threshold determination is that the threshold should be able to maximize the feature difference between the target and the background (17).

Variance is a statistical measure of the uniformity of distribution. Therefore, the segmentation that maximizes the variance between classes means the smallest misclassification probability.

If T is regarded as the segmentation threshold of target and background, the proportion of foreground (target) region to image is denoted as w_0_, and the average gray level is u_0_. The proportion of background region to the image is denoted as w_1_, the average gray level is u_1_, the total average gray level of the image is U, and the variance of foreground and background image g(T) is as follows:


(1) $$\begin{array}{l} u=w_{0}\times u_{0}+w_{1}\times u_{1} \end{array}$$

(2) $$\begin{array}{l} g\left(T\right)=w_{0}\times {\left(u_{0}-u\right)}^{2}+w_{1}\times {\left(u_{1}-u\right)}^{2} \end{array}$$

Since under this method, the image is only treated as foreground and background overlay, there are


(3) $$\begin{array}{l} w_{0}{+w}_{1}=1 \end{array}$$

Equations (1)–(3) can be obtained simultaneously


(4) $$\begin{array}{l} g\left(T\right)=w_{0}\times w_{1}\times {\left(u_{0}-u_{1}\right)}^{2} \end{array}$$

To ensure that g(T) is the largest, T is selected as the desired. Notice that this formula w_0_ is symmetric about w_1_, so w_0_ can be set as the partition less than the threshold value, that is, the foreground represents the region s_0_ occupied by all gray values in the image S less than the threshold value. u_0_ represents the average gray level of s_0_ region.


(5) $$\begin{array}{l} w_{0}=\frac{s_{0}}{s} \end{array}$$

(6) $$u_{0}=\frac{\sum s_{0}}{s_{0}}$$

Here, s_1_ is the same as u_1_. And then one obtains g(T) as a function of T and then you invert the maximum value of T for g of T.

##### Threshold iteration method

The main idea of threshold iteration method is that the mean sum of two parts A and B after image segmentation basically remains stable (18). In other words, with the iteration, the final convergence value of [mean(A)+mean(B)]/2 is taken as the segmentation threshold. The specific method is as follows:

(1) Select an initial threshold T;
(2) The given image is divided into two groups of images by threshold T, denoted as R1 and R2;
(3) Calculate the mean values of R1 and R2 μ1 and μ2;
(4) Select a new threshold T, and T= (μ1 + μ2) /2;
(5) Repeat steps (2) to (4) until the difference of T for two consecutive times is less than a preset value.

**Figure 3:**
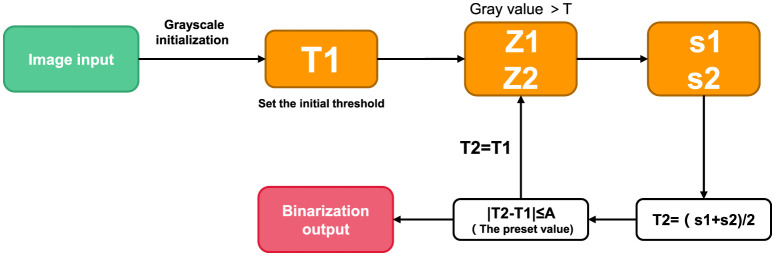
Program flow chart of threshold iteration method.

On this basis, in the case that the target region is equal to the background region, the initial threshold T is set as the average gray value of the overall image, so as to accelerate the convergence rate. In the case of large difference between target and background area, the initial threshold T is set as the intermediate value between the maximum gray scale and the minimum gray scale. The default values depend on the image size, grayscale distribution, and calculation time required, but are generally small and accurate. Its construction is shown in Figure 3.

##### The maximum entropy method

The principle of image maximum entropy threshold segmentation: make the selected threshold to segment the target area and background area of image gray statistics of the two parts of the maximum information (19).

The area whose gray value is ≤T is S0, and the rest of the image is S1. Let PI represent the probability of gray value being I, and PT represent the sum of gray value probabilities from 0 to T. H (S) represents the information entropy of the region.


(7) $$\begin{array}{l} P_{T}=\overset{T}{\underset{i=0}{\sum }}p_{i} \end{array}$$

(8) $$\begin{array}{l} H\left(S_{0}\right)=-\overset{T}{\underset{i=0}{\sum }}\frac{p_{i}}{P_{T}}\ln\frac{p_{i}}{P_{T}} \end{array}$$

(9) $$\begin{array}{l} H\left(S_{1}\right)=-\overset{T}{\underset{i=T+1}{\sum }}\frac{p_{i}}{{1-P}_{T}}\ln\frac{p_{i}}{{1-P}_{T}} \end{array}$$

By traversing the gray value, T that maximized H(S0)+H(S1) was found.

#### The difficulty of threshold segmentation

The difficulties of threshold segmentation are as follows: (1) Before image segmentation, the number of regions generated by image segmentation cannot be determined; (2) Determination of threshold value, because the determination of threshold value directly affects the accuracy of segmentation and the correctness of description analysis of the segmented image.

### UU-NET network

#### UU-NET

In 2015, Ronneberger et al. trained the U-NET model based on convolutional neural network with a small number of training samples and won the ISBI Cell Tracking Challenge. The network is called U-NET because it is shaped like the letter “U”. Subsequently, U-NET network obtained excellent segmentation results in medical images of the heart.

**Figure 4:**
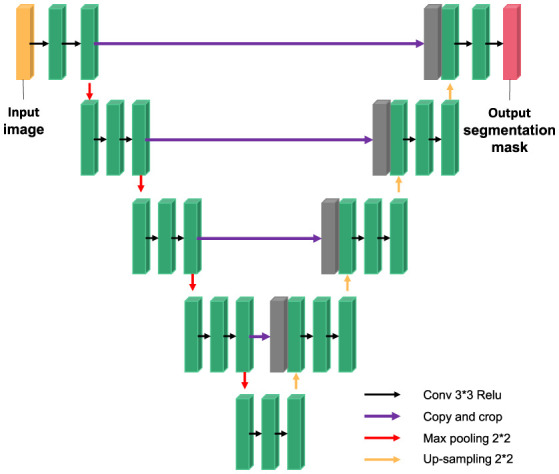
U-NET network model.

The U-NET model consists of a downsampling encoder and an upsampling decoder, as shown in Figure 4. The structure is simple and easy to understand. The contraction process of the model is the down-sampling process. The author extracts features through multiple 3^*^3 convolution layers and uses the maximum pooling of 2^*^2 for down-sampling (20). In the expansion process of the model, that is, the upsampling process, the feature graph of the previous stage is firstly upsampled, and then the feature graph of the contraction process is splice and further refined by jumping connection. Finally, the channel number mapping of the feature map is scaled down to the segmented category number through the 1^*^1 convolution layer. U-net has been widely used in the field of medical image segmentation because of its simple structure, easy transformation and adjustment, and its high ability of obtaining context information and precise positioning.

#### Data preprocessing and data amplification

##### Data standardization

Due to the differences of acquisition equipment and conditions in various research institutions, there are differences in brightness and contrast of cardiac MRI images. The essence of neural networks is to learn by distributing data. If there is a big difference between the training data and the experimental data, the generalization ability of the model will be greatly weakened. To solve this problem, it is often required to normalize the input data.

On this basis, cardiac short-axis MRI was processed to obtain a normalized data with 0 pixel mean, 1. On this basis, a normalization algorithm based on maximum normalization is proposed.


(10) $$\begin{array}{l} x=\frac{x-x_{mean}}{x_{\max}-x_{min}} \end{array}$$

Where x_mean_ represents the average value of the required data, x_max_ represents the maximum value of the required data, and x_min_ represents the minimum value of the required data.

##### Data enhancement

However, due to the high cost of image acquisition, social ethical privacy and high human cost of professionals, many medical image research based on deep learning cannot obtain enough data for training or verification. However, in the case of small samples, there is the problem of over-fitting. The data intensification methods used in this paper include −0.2–0.2 random scale, −5 to +5% random direction shearing, −5 to +5% contrast correction, etc.

#### UU-NET model structure

Based on the improvement of U-NET model, this paper designs a UU-NET model which is more suitable for medical image segmentation. In this paper, u-shaped upper and lower sampling modules are constructed by using residual theory, which are used as encoders and decoders of the model. Then, the modules are interconnected to form multiple paths from input to output to increase the information transmission capability of the model.

##### Residual module

The depth of neural network largely determines image classification and computer vision tasks. Theoretically, the higher the depth of the network, the better its fitting effect. But that's not true. This is because with the increase of network depth, the gradient will gradually weaken, thus affecting the segmentation of the network. In this paper, we use ResNet to delay gradient extinction in network propagation.

**Figure 5:**
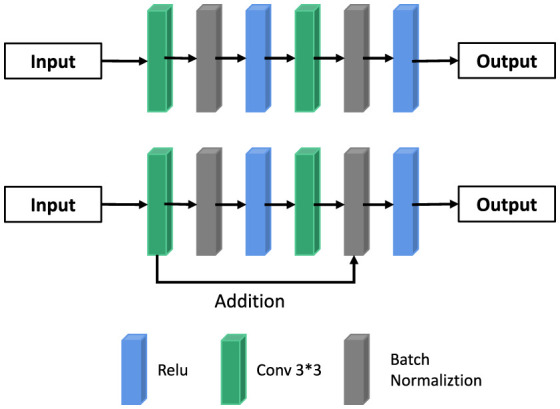
Ordinary convolution block and residual convolution block.

The mathematical principle of ResNet is as follows: Assume that the mapping of input X to output Y is H, that is, Y = H(X); After input X passes through multiple nonlinear layers, the result Y satisfies another mapping as F, that is, Y = F(X), where F(X) = H(X) -x between F and H satisfies. Thus, the original mapping H is represented as F(X) + X. In the feedforward neural network, the required mapping H can be realized through the connection mode of “layer hopping,” as shown in Column B in Figures 2, 3. The UU-NET model in this paper uses this residual idea to construct convolution blocks in U-NET as shown in Figure 5. The essence is to replace ordinary convolution modules with residual convolution modules and apply batch regularization rules to model training.

##### Sampling module

**Figure 6:**
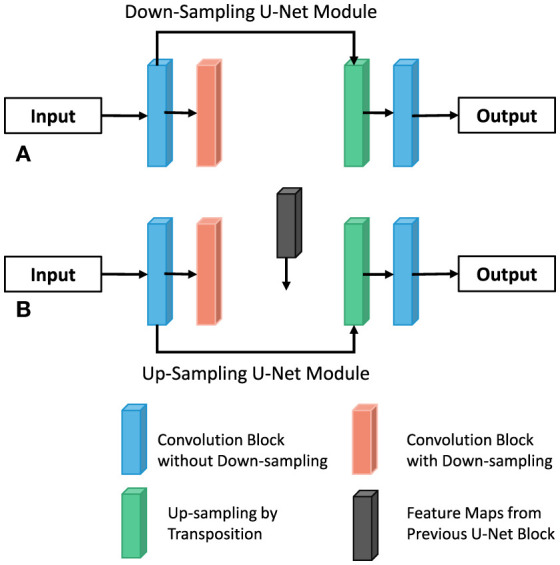
Modules of two UU-NET models. **(A)** Down-Sampling. **(B)** Up-Sampling of the U-Net module.

By connecting the intermediate result of the former U-NET decoder to the latter U-NET encoder through Addition, the latter can be supplemented with additional information on the feature graph of each scale. At the same time, this method can construct multiple paths to transmit information, and each path is equivalent to an FCN variant. Therefore, it has the potential to capture more complex features and produce higher accuracy. Similarly, in order to construct multiple paths, this paper constructs two types of U-NET modules, which are respectively used for the outer U-shaped encoder (column A and decoder in Figure 6 and column B in Figure 6 for the design of UU-NET model.

##### Overall model structure

**Figure 7:**
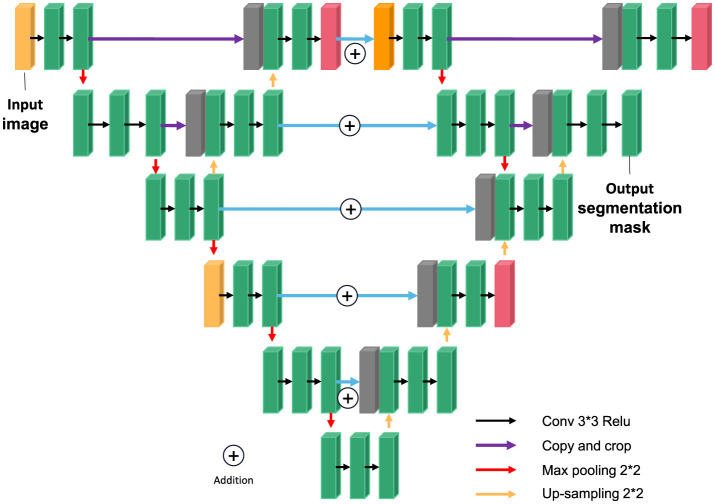
Overall structure of UU-NET network.

Through the construction of two types of U-NET modules, referred to as external U encoder module and external U decoder module, the UU-NET model is designed. The “hop layer” in the U-NET module is connected by Concatenation. The smallest feature graph in the outer U encoder module will be the input of the lower outer U encoder module. U decoder module and external U encoder module on the size of the corresponding figure for Addition (Addition) operation, namely “U” encoder module and the intermediate results produced by decoding part of the “U” decoder module coding and some intermediate results, so the latter on the characteristics of various scale chart to get the additional information added, at the same time, Added multiple paths from input to output. Compared with LadderNet, this paper not only uses the residual convolution block to increase the depth of the outer U-shaped structure, but also constructs more information transmission paths through the double-layer structure, that is, more FCN variants are added. As shown in Figure 7, the outer layer of the normal U-Net model is connected by residual convolution blocks, thus forming a double-layer U model. It is equivalent to regard each neural network of U-NET model as a U-NET model.

### Deconvolution neural network

**Figure 8:**
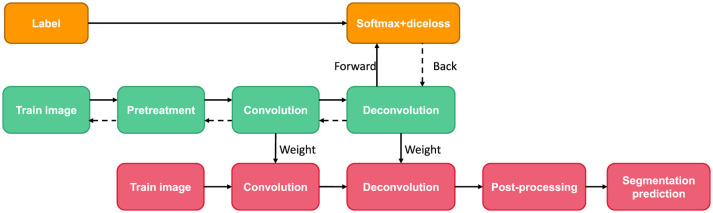
Deconvolution neural network framework used in this paper.

Deep deconvolution network is a supervised learning method, which includes training stage and test stage (as shown in Figure 8). Ten times in the training phase, this paper uses the method of cross validation, the magnetic resonance image segmentation and manual real image as the training sample input of neural network, through the forward and reverse transmission, iteratively weight training network model, and with the other five patients image of the sample as the validation sample, for training the model provide supervision and guidance. Finally, a Softmax classifier was trained and Dice loss function was optimized to obtain the classification probability map of the whole image. In the test stage, the test image is input into the trained network model after contrast adjustment processing, and the final test image segmentation result is obtained through a forward propagation calculation.

#### Structure and parameters of deconvolution networks

**Figure 9:**
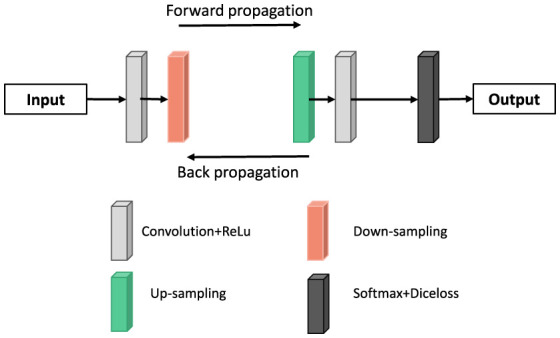
Structure diagram of deconvolution network.

Figure 9 shows the network architecture referenced here. In order to facilitate network input, all input images are uniformly divided into 3 × 2,244 × 224 pixels. In convolutional networks, we use a similar structure to FCN, and replace all the final connection layers with a convolutional layer. The convolutional network model includes five convolution layers, five maximum layers and two convolution layers. The convolution method is an overlapping method, that is, one or two successive convolution layers are placed behind each convolution layer. In the network, all convolution kernels are small Windows 3 × 3 and 1 step long. In order to keep the size of the feature graph consistent before and after the convolution operation, a numerical value “0” of 1 is added to the edge of the input feature graph. Overlapping convolutional layer can not only improve the depth of the network, learn the parameters of the network, but also effectively prevent over-fitting.

In the deconvolution network part, the mirror structure of the convolutional network is used to reconstruct the input object. Therefore, multilayer deconvolution can also obtain different morphological details like convolutional networks. In the convolutional network model, low-level features can reflect the overall roughness of the target, such as target position and rough shape, while higher-level features have classification characteristics and contain the details of the target.

#### Dice depletion layer

The image is trained into the network, and then the software mapping layer is used to get the classification map with the same resolution as the initial input, so as to reflect the possibility that each pixel point is the foreground or background. On this basis, the loss function is optimized by using the classification method based on actual tags, so as to obtain the final weight of the convolutional network. In the learning process, due to the strong learning characteristics of the network, the usual loss function tends to fall into local minimization, resulting in the detection of future regions. In order to improve the response curve after classification, a new target loss function D was established by Dice similarity coefficient, which made the predicted response curve and the actual segmentation result have the maximum similarity.


(11) $$D=\frac{2\sum_{i}^{N}p_{i}g_{i}}{\sum_{i}^{N}p_{i}^{2}\sum_{i}^{N}g_{i}^{2}}$$

Where, p_i_ and g_i_ represent the value of the ith pixel in the prediction graph and the real segmentation graph, respectively. We take the derivative of this


(12) $$\frac{\partial D}{\partial p_{i}}=2\left[\frac{g_{i}\left(\sum_{i}^{N}p_{i}^{2}+\sum_{i}^{N}g_{i}^{2}\right)-2p_{i}\left(\sum_{i}^{N}p_{i}g_{i}\right)}{{\left(\sum_{i}^{N}p_{i}^{2}+\sum_{i}^{N}g_{i}^{2}\right)}^{2}}\right]$$

The partial derivative is used for parameter updating in back propagation. Experimental results show that Dice loss function has better performance than ordinary loss function.

### Evaluation standard

#### Parameters of the standard

On this basis, DSC and Hausdorff distance (HD) were used to evaluate the two methods quantitatively. DSC algorithm performs similarity analysis in the range of two contour lines. The point set within the range of two contour lines represented by A and B is defined as follows


(13) $$\begin{array}{l} DSC\left(A,B\right)=\frac{2|A\cap B|}{\left|A|+|B\right|} \end{array}$$

HD reflects the maximum difference between the two contour point sets and is defined as


(14) $$\begin{array}{l} HD\left(A,B\right)=max\left(h\left(A,B\right),h\left(B,A\right)\right) \end{array}$$

Among them


(15) $$h(A,B)=\overset{\text{max min}}{{\text{a}}_{\text{i}}\varepsilon {\text{Ab}}_{\text{i}}\varepsilon \text{B}}||a_{i}-b_{i}||$$

(16) $$||a_{i}-b_{j}||=\sqrt{{\left(x_{a_{i}}-x_{b_{j}}\right)}^{2}+{\left(y_{a_{i}}-y_{b_{j}}\right)}^{2}}$$

Smaller HD values mean higher segmentation accuracy.

## Results

### The experimental analysis

A total of 150 patients were enrolled in the Department of CT/MRI at the Second Affiliated Hospital of Fujian Medical University, 8 to 15 groups for each. Each sequence consisted of a 20-frame cardiac cycle, consisting of 15,000 artificially segmented images, 12,000 images as training samples, and 3,000 images as experimental samples. From the initial reading of cardiac MRI data, to the normalization described in the previous, to the normalization and data enhancement of image data, and then, according to the ROI area center and ROI detection results, the image data were divided into 128 × 128 pieces, clustered in the ROI center, namely the left ventricle, and input to the ROI center, namely the left ventricle. Compared with the original cardiac MRI slice with average spatial resolution of 235–263 voxel/slice, the GPU storage capacity was reduced from more than 10 GB to <6 GB by using the same model for training. This method uses ROI to extract 128 × 128 images and reduces unnecessary information while maintaining the left exterior shape.

The segmentation results of region growing image differ greatly when the gray difference threshold is different. When the threshold was small, the whole myocardium could not be separated. When the threshold is large, other surrounding tissues are incorrectly separated. However, the determination of threshold depends on the determination in advance, so it cannot adapt to the changeable conditions of cardiac MR images.

Since other tissues outside the myocardium are similar to the myocardium in gray scale, threshold segmentation method only uses gray level to determine whether pixels belong to segmentation target, without utilizing relevant spatial information. Therefore, tissues near the myocardium with similar gray scale are often segmented together. Threshold method is not effective in ventricular segmentation of cardiac MRI.

The deep learning image segmentation method is relatively ideal, but the deconvolution neural network is sometimes over-sharpened.

**Figure 10:**
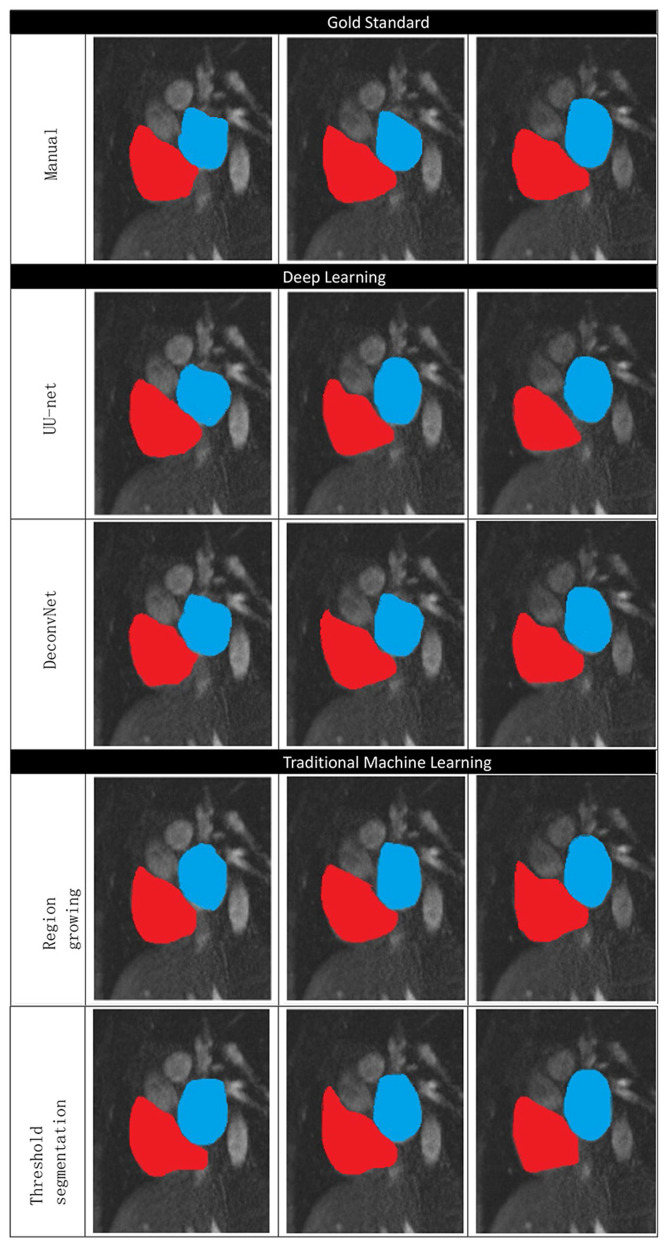
Comparison of traditional methods, deep learning, and manual segmentation in atrial segmentation.

Figure 10 shows the manual segmentation and traditional image segmentation methods (region growth, threshold segmentation) and deep learning image segmentation method (UU-NET, deconvolution neural network), where red is right atrium (RA) and blue is left atrium (LA).

### The experimental results

Table 1 lists the performance of the relevant methods mentioned in this article. As can be seen from the table, deep learning image segmentation algorithm has obvious advantages over traditional methods, while DSC obtained by the algorithm in this paper is slightly higher than deconvolution neural network, which means that the image segmentation completed in this paper is more accurate in atrial coverage. At the same time, it has obvious advantages in HD, which means that the set of contour points in this paper is closer to the standard, that is, the probability of sharpening is lower. At the same time, we should also realize that the processing time of computer-aided calculation is far shorter than that of manual segmentation (15–20 min) by professional doctors, which indicates that the application of deep learning in image segmentation is a general trend.

**Table 1:** Result data of each method on validation set.

**Methods**	**DSC/%**	**HD/mm**	**Time/min**
Region growing	83.4	9.3	4
Threshold segmentation	86.7	8.5	6
Deconvolution neural network	95.8	5.5	1.4
UU-NET	96.7	1.3	0.4

## Discussion

Segmentation of medical image is an important and difficult problem in clinical diagnosis (21). Accurate segmentation, identification and analysis can be used for qualitative and quantitative analysis of target organs and tissues in clinical practice, improve the accuracy and reliability of diagnosis, and indirectly improve the survival of patients (22). MRI has been widely used in the diagnosis and evaluation of many important diseases including cardiovascular diseases (23). It is widely used. In order to solve the problem of ventricular segmentation in MRI, a new deep deconvolution neural network is used for segmentation, and good results are obtained in the experiment. However, on the basis of in-depth analysis of deep learning technology, existing problems are found and possible directions for further research are proposed:

1. Reliable data sets. First, due to the high cost, there are relatively few relevant data sets that can be used for deep learning modeling. Although common data expansion means can be used to expand, the expansion cannot reflect the sample distribution in the real world, which may have hidden dangers for clinical use. Second, there is no unified standard, because the equipment of different research institutions is not consistent, the data quality, code, scale and so on are not consistent.
2. Expand the application scope. The current application scope of the model in this paper is 2D cardiac MRI, which can be considered for segmentation of other medical images, such as CT, ultrasound and X-ray. Segmentation of medical images of other parts, such as head, neck, chest, etc. The 3D heart model was segmented. In order to better present relevant information to doctors from multiple fields.
3. Improve noise resistance. Because of the particularity of medical image acquisition, shadow and noise are common phenomena. There is still room to improve the noise resistance of the model used in this paper. The subsequent optimization may be carried out by adopting a more suitable preprocessing method and a more accurate loss function.
4. Stereotypes. The types of training sets are also different, which leads to poor generalization performance of the models. Increasing the diversity of data is key to solving this problem. However, one is the lack of credible data in the above data, and the other is that some cases are very rare in the history of human medicine.

Because of the complexity of medical images and gradient fuzzy boundaries, need more high resolution information, after join operations directly from the encoder to the decoder with height on the high-resolution information can provide the division with the fine features, such as gradient, and in view of the left ventricle segmentation data sets have labeled images is small in the segmentation problem such as low precision, learning difficulties, Deep learning in medicine has a long way to go (24–26).

## Conclusion

The segmentation task of atrial image is realized by using the latest deep learning technology. In order to achieve pixel-by-pixel segmentation of images, this paper proposes an embedded UU-NET model with dual U structure, which is used to segment the left atrium of MRI data preprocessed by subjective screening, data amplification and image enhancement. Experimental verification shows that this design method improves the segmentation accuracy of left and right atrial in MRI. The experimental results show that the segmentation accuracy of UU-NET model has exceeded that of partial deep learning method. Further improvements will be made under the guidance of experts.

## Data availability statement

The original contributions presented in the study are included in the article/supplementary material, further inquiries can be directed to the corresponding author/s.

## Ethics statement

The studies involving human participants were reviewed and approved by the Second Affiliated Hospital of Fujian Medical University. The patients/participants provided their written informed consent to participate in this study.

## Author contributions

Study design: YW and S-TL. Data interpretation: JH and Q-QL. Manuscript production and takes responsibility for the integrity of the data analysis: YW and Y-ZL. All authors contributed to the article and approved the submitted version.

## Conflict of interest

The authors declare that the research was conducted in the absence of any commercial or financial relationships that could be construed as a potential conflict of interest.

## Publisher's note

All claims expressed in this article are solely those of the authors and do not necessarily represent those of their affiliated organizations, or those of the publisher, the editors and the reviewers. Any product that may be evaluated in this article, or claim that may be made by its manufacturer, is not guaranteed or endorsed by the publisher.
